# 1-hour post-load plasma glucose: a reliable marker of insulin resistance in type 2 diabetes, as determined by the TyG index

**DOI:** 10.3389/fendo.2025.1671040

**Published:** 2026-01-09

**Authors:** Qingyu Guo, Li Lv, Xinyi Yang, Min Chen, Chen Chen, Ting Chen, Lili Xu, Qiuyue Shen, Ping Gu, Jiaqing Shao

**Affiliations:** Department of Endocrinology, Jinling Hospital, Affiliated Hospital of Medical School, Nanjing University, Nanjing, China

**Keywords:** 1-hour post-load plasma glucose, 2-hour post-load plasma glucose, insulin resistance, triglyceride-glucose, type 2 diabetes mellitus

## Abstract

**Objective:**

This study aimed to investigate the correlation between 1-hour postprandial glucose (1h-PG) and the triglyceride-glucose (TyG) index in type 2 diabetes mellitus (T2DM) subjects and to evaluate the predictive utility of 1h-PG for insulin resistance (IR).

**Research design and methods:**

Based on the criteria of 1h-PG ≥ 8.6mmol/L and 2h-PG ≥ 11.1mmol/L from the 125-gram standard steamed buns meal test, 1835 T2DM individuals were categorized as follows: Group 1: 1h-PG < 8.6mmol/L; Group 2: 1h-PG ≥ 8.6mmol/L and 2h-PG < 11.1mmol/L; Group 3: 1h-PG ≥ 8.6mmol/L and 2h-PG ≥ 11.1mmol/L. The severity of IR was assessed using the TyG index, with thresholds of > 8.81 for males and > 8.73 for females. Clinical and laboratory parameters, including glucose, C-peptide, insulin and lipid profiles were analyzed. Statistical analyses included Spearman’s correlation, logistic regression, and receiver operating characteristic (ROC) curve analysis.

**Results:**

1h-PG exhibited a more pronounced positive correlation with the TyG index (*r* = 0.273) than 2h-PG (*r* = 0.173). A statistically significant increase in the TyG index was observed in Group 2 compared with Group 1. Although Group 3 showed a trend toward a higher TyG index compared to Group 2, the difference was not statistically significant. The higher insulin resistance group exhibited significantly higher 1h-PG levels compared to the lower insulin resistance group. Logistic regression confirmed that the association between 1h-PG and the TyG index was stronger (*OR* = 1.170, 95% *CI*: 1.133–1.209, *P* < 0.001) than that of 2h-PG (*OR* = 1.091, 95% *CI*: 1.062–1.121, *P* < 0.001). ROC analysis revealed that 1h-PG achieved a higher AUC for predicting IR than 2h-PG (0.629 *vs*. 0.589, *P* < 0.0001).

**Conclusion:**

Among T2DM subjects, 1h-PG shows a stronger association with the TyG index than 2h-PG. These findings support the potential of 1h-PG as a more sensitive and cost-effective marker for early identification of insulin resistance, offering clinical utility in diabetes diagnosis and management.

## Introduction

Insulin Resistance (IR): Also referred to as “insulin insensitivity”, is primarily defined by a diminished ability of peripheral tissues to uptake and utilize glucose, particularly skeletal muscle, fat tissue, and the liver, resulting in an impaired response to insulin. This pathophysiological state is not only recognized as the “common soil” for multiple metabolic disorders such as type 2 diabetes mellitus (T2DM), atherosclerotic cardiovascular disease (ASCVD), non-alcoholic fatty liver disease (NAFLD) and polycystic ovary syndrome (PCOS), but also contributing significantly in the pathophysiology of certain rare diseases, like insulin autoimmune syndrome (1). Despite the clinical importance of IR, its early identification in patients remains challenging. Traditional indices such as HOMA-IR require fasting insulin measurements, which are often costly and not routinely available in clinical practice. Recent studies, both domestically and internationally, have identified the triglyceride-glucose (TyG) index as a robust, trustworthy, and clinically feasible marker for assessing IR (2). The TyG index, which is calculated using fasting triglyceride and glucose levels, was initially proposed in 2008 by Simental-Mendía et al. in a cross-sectional study of healthy individuals. The TyG index is derived using the following equation: Ln [fasting triglycerides (mg/dL) × fasting plasma glucose (mg/dL)/2] (3). Multiple studies have demonstrated the predictive and diagnostic value of the TyG index across various insulin resistance-related conditions, highlighting its potential utility in both clinical and research settings.

Recent guidelines, including the 2024 International Diabetes Federation Position Statement, have recommended the inclusion of 1-hour post-load plasma glucose (1h-PG) as a diagnostic marker for intermediate hyperglycemia (IH) and T2DM (4). Accumulating evidence suggests that elevated 1h-PG is strongly associated with adverse cardiovascular outcomes. For example, Rong et al. reported that among high cardiovascular risk individuals, 1h-PG ≥11.1 mmol/L more reliably predicted cardiovascular mortality than 2h-PG, with a dose-dependent increase in event and mortality risk (5). Long-term cohort studies, including a 33-year follow-up of 1,945 non-diabetic Israelis and the 39-year MPP study, further demonstrated that 1h-PG ≥8.6 mmol/L in individuals with normal glucose tolerance (NGT) was associated with significantly higher risks of myocardial infarction, fatal cardiovascular disease, and all-cause mortality (6, 7). Notably, incorporating 1h-PG into conventional clinical risk models significantly improved predictive performance for both cardiovascular and all-cause outcomes.

Beyond its cardiovascular relevance, emerging evidence indicates that elevated 1h-PG reflects compromised insulin sensitivity and impaired pancreatic β-cell function in NGT individuals (8–10). Despite these insights, the relationship between 1h-PG and IR in patients already diagnosed with T2DM remains poorly understood, representing a critical knowledge gap. Therefore, the primary aim of this study was to investigate whether 1h-PG exhibits a stronger association with the TyG index, a validated surrogate of IR, compared to 2h-PG, in a T2DM population. Addressing this question may provide valuable evidence for early identification of IR and inform clinical strategies for risk stratification in T2DM patients.

## Research design and methods

### Participants

Exactly 1835 subjects already diagnosed as T2DM hospitalized at the endocrinology department of the Jinling Hospital of Nanjing University during October 2017 to October 2024 were enrolled. All participants met the diagnostic criteria for T2DM as defined by the World Health Organization (WHO) in 1999 (11). The exclusion criteria were as follows: (1) other types of diabetes; (2) presence of acute diabetic complications; (3) acute stress conditions like severe infections, trauma, or surgery; (4) critical cardiovascular or cerebrovascular diseases; (5) comorbid hematologic disorders or infectious diseases; (6) history of narcotic or psychotropic drug use or recent alcohol intoxication; and (7) malignancy or pregnancy.

### Methods

1. Physical and laboratory examination: Clinical characters, such as age, sex, and duration of diabetes, were recorded. Height, weight, systolic blood pressure (SBP), and diastolic blood pressure (DBP) were measured, and body mass index (BMI) was calculated. Blood samples were gathered overnight fasting for fasting plasma glucose (FPG), creatinine, triglycerides (TG), low-density lipoprotein cholesterol (LDL-C), alanine aminotransferase (ALT), and aspartate aminotransferase (AST). Glycated hemoglobin (HbA1c), fasting C-peptide and fasting insulin (FINS) levels were measured using an electrochemiluminescence immunoassay (IMMULITE 2000 XPi, Siemens, Germany). Insulin resistance indices, including HOMA-IR, homeostasis model assessment of β-cell function (HOMA-β), quantitative insulin sensitivity check index (QUICKI), and triglyceride-glucose (TyG) index, were calculated using the following formulas: HOMA-IR = [FINS (mU/L) × FPG (mmol/L)]/22.5; HOMA-β = [20 × FINS (mU/L)]/[FPG (mmol/L) - 3.5]; QUICKI = 1/[log FINS (μU/mL) + log FPG (mg/dL)]; TyG = ln [TG (mg/dL) × FPG (mg/dL)/2]; and TyG-BMI = TyG × BMI. All enrolled patients underwent a standardized 125g steamed buns meal test during hospitalization, which serves as an alternative to the oral glucose tolerance test (OGTT) for diabetes diagnosis (11). Prior to the test, participants commenced an overnight fast starting at 22:00 the preceding evening. Serum C-peptide, insulin, and glucose levels were quantified at 0, 0.5, 1, 2, and 3 hours postprandially.
2. Classification: Based on the criteria of 1h-PG ≥ 8.6mmol/L and 2h-PG ≥ 11.1mmol/L from the 125-gram standard steamed buns meal test, participants were categorized as follows: Group 1: 1h-PG < 8.6mmol/L(n=60); Group 2: 1h-PG ≥ 8.6mmol/L and 2h-PG < 11.1mmol/L(n=116); Group 3: 1h-PG ≥ 8.6mmol/L and 2h-PG ≥ 11.1mmol/L(n=1659). (2) A large-scale study encompassing 30,291 Chinese participants established TyG index cutoff values of 8.81 for males and 8.73 for females as diagnostic thresholds for insulin resistance, with values exceeding these thresholds indicating the higher insulin resistance(derived from a study of Chinese adults, reference 12) (12). According to these criteria, 1,835 participants were categorized into the lower insulin resistance and the higher insulin resistance groups. The lower insulin resistance group included 566 individuals, while the higher insulin resistance group comprised 1269 individuals.
3. Statistical analyses were conducted using SPSS software (V.26, IBM-Armonk), R4.3.1 (http://www.r-project.org/) and MedCalc Statistical Software version 15.8 (MedCalc Software bvba, Ostend, Belgium). When continuous variables were normally distributed, we used mean ± standard deviation (x¯ ± s) for their presentation. Independent samples t-tests were applied for pairwise comparisons, while one-way analysis of variance was implemented for comparisons across three or more groups, with *post hoc* pairwise comparisons adjusted by the Bonferroni test. When continuous variables were non-normally distributed, we used median (interquartile range, IQR) for their presentation, employed nonparametric tests: Mann-Whitney U for pairwise comparisons and Kruskal-Wallis for multiple-group comparisons, with *post hoc* pairwise comparisons adjusted by the Bonferroni test. Categorical variables were presented as frequencies (percentages), and comparisons between groups were performed using the chi-square test. Spearman’s rank correlation analysis was employed to evaluate bivariate associations between 1h-PG, 2h-PG and various indices of insulin resistance. The distribution of the TyG index across three groups was visualized using R 4.3.1. Univariate binary logistic regression analysis was employed to further investigate the relationships between 1h-PG, 2h-PG, and the TyG index. The predictive ability of 1h-PG and 2h-PG for insulin resistance (assessed by the TyG index) in patients with T2DM was evaluated using receiver operating characteristic (ROC) curve analysis, with the area under the curve (AUC) calculated. The comparison of AUCs was performed using MedCalc software. A two-tailed *P*-value < 0.05 was considered statistically significant.

## Results

1. Association between 1h-PG, 2h-PG and indices of insulin resistance.

As shown in **Table 1**, we used Spearman’s correlation analysis to evaluate the relationship between 1h-PG, 2h-PG, and indices of insulin resistance. The results revealed that 1h-PG was positively correlated with the TyG index and HOMA-IR (*r* = 0.273 and 0.049, respectively), while it was negatively correlated with HOMA-β and QUICKI (*r* = -0.369 and -0.049, respectively). Similarly, 2h-PG was positively correlated with the TyG index and HOMA-IR (*r* = 0.173 and 0.082, respectively), whereas it showed negative correlations with HOMA-β and QUICKI (*r* = -0.322 and -0.082, respectively). Notably, the correlation between 1h-PG and the TyG index was stronger than that between 2h-PG and the TyG index, indicating a more pronounced positive association for 1h-PG.

**Table 1:** The correlation of 1-hour post-load plasma glucose, 2-hour post-load plasma glucose and parameters of insulin resistance.

Variables	*r/P*	TyG	HOMA-IR	HOMA-β	QUICKI
1-hour post-load plasma glucose	*r*	0. 273	0.049	-0.369	-0.049
	*P*	<0.001	0.036	<0.001	0.036
2-hour post-load plasma glucose	*r*	0.173	0.082	-0.322	-0.082
	*P*	<0.001	0.000	<0.001	<0.001

TyG, Triglyceride glucose; HOMA-IR, homeostasis model assessment of insulin resistance; HOMA-β, homeostasis model assessment of β-cell function; QUICKI, quantitative insulin sensitivity check index.

2. Based on the criteria of 1h-PG ≥ 8.6mmol/L and 2h-PG ≥ 11.1mmol/L from the 125-gram standard steamed buns meal test, participants were categorized as follows: Group 1: 1h-PG < 8.6mmol/L; Group 2: 1h-PG ≥ 8.6mmol/L and 2h-PG < 11.1mmol/L; Group 3: 1h-PG ≥ 8.6mmol/L and 2h-PG ≥ 11.1mmol/L. As shown in **Table 2**, A comparative analysis of baseline clinical characteristics and insulin resistance indices was performed across three groups. This study included all of 1835 individuals, with 60 in Group 1, 116 in Group 2, and 1659 in Group 3. Compared to Group 1, Group 2 exhibited a higher proportion of males, elevated 1-hour and 2-hour postprandial C-peptide levels during the steamed buns meal test, and increased the TyG index and TyG-BMI values, while no significant differences were observed in HOMA-IR, HOMA-β, or QUICKI. In contrast, when comparing Group 3 to Group 2, participants were characterized by older age, longer disease duration, higher HbA1c levels, and lower proportion of males, as well as reduced BMI, serum creatinine (Scr), fasting C-peptide, 1-hour postprandial C-peptide, 2-hour postprandial C-peptide, and HOMA-β levels. However, no significant differences were noted in FINS, TyG-BMI, HOMA-IR, or QUICKI between Group 2 and Group 3. Although a trend toward higher TyG index was observed in Group 3, this difference did not reach statistical significance. Overall, these findings indicate that 1h-PG exhibits a stronger association with the TyG index than 2h-PG, supporting its potential as a more sensitive marker of insulin resistance in this population.

**Table 2:** The Clinical characteristics among different groups.

Variables	Group1 (1h-PG < 8.6)	Group2 (1h-PG ≥ 8.6and 2h-PG < 11.1)	Group3 (1h-PG ≥ 8.6and 2h-PG > 11.1)	*x2/t/z*	*P*
*N*	60	116	1659	–	–
Age (y)	53.88 ± 12.66	50.56 ± 11.36	54.57 ± 12.77 ‡	5.461	0.004
Diabetes duration (y)	4.50 (1.19, 12.00)	4.00 (1.00, 9.00)	7.00 (2.00, 13.00) ‡	15.394	<0.001
Male (n, %)	(44, 73.33)	(87, 75.00) †	(1089, 65.64) ‡	3139.68	<0.001
SBP (mmHg)	134.82 ± 19.85	133.09 ± 15.82	134.58 ± 17.19	0.422	0.656
DBP (mmHg)	79.77 ± 10.58	81.43 ± 10.18	80.70 ± 10.48	0.519	0.595
BMI(kg/m^2^)	25.17 ± 4.85	25.96 ± 3.24	25.27 ± 3.60 ‡	2.005	0.135
ALT(U/L)	21.00(14.00, 33.00)	18.50(13.00, 28.75)	19.00(13.00, 28.00)	0.777	0.678
AST(U/L)	18.00(14.00, 23.00)	16.00(14.00, 22.75)	17.00(14.00, 22.00)	0.878	0.645
LDL (mmol/L)	2.69 ± 0.97	2.63 ± 0.84	2.74 ± 1.09	0.630	0.533
Scr (µmol/L)	61.55(53.40, 78.38)	63.50(50.00, 73.00)	57.00(46.00, 68.00) ‡	15.960	<0.001
C peptide (ng/mL)	1.60(0.83, 2.55)	1.85(1.19, 2.66)	1.34(0.77, 2.05) ‡	28.490	<0.001
FINS (mIU/L)	9.58(3.33, 16.05)	7.54(4.10, 13.40)	6.34(2.61, 13.90)	4.297	0.117
1-h C peptide (ng/mL)	3.09(1.07, 5.00)	4.96(3.44, 6.98) †	2.75(1.66, 4.21) ‡	76.129	<0.001
2-h C peptide (ng/mL)	4.25(2.21, 7.25)	6.51(4.93, 9.35) †	4.03(2.42, 6.11) ‡	63.668	<0.001
TyG	8.67(8.11, 9.11)	9.10(8.56, 9.56) †	9.16(8.68, 9.69)	26.086	<0.001
TyG-BMI	214.16(184.38, 261.57)	227.80(211.35, 259.67) †	231.50(203.40, 259.63)	4.760	0.093
HOMA-IR	2.14(0.77, 4.59)	1.84(1.11, 3.35)	2.24(1.00, 5.04)	2.547	0.280
HOMA-β	100.33(37.98, 273.50)	63.38(33.37, 127.09)	32.13(14.29, 79.37) ‡	59.495	<0.001
QUICKI	0.34(0.31, 0.40)	0.35(0.32, 0.38)	0.34(0.30, 0.38)	2.547	0.280
HbA1c(%)	7.10(6.03, 9.90)	6.60(6.00, 7.78)	8.80(7.50, 10.40) ‡	128.024	<0.001

a SBP, systolic blood pressure; DBP, diastolic blood pressure; BMI, body mass index; ALT, Alanine aminotransferase; AST, Aspartate aminotransferase; LDL, low‐density lipoprotein; FINS, fasting insulin; SCr, serum creatinine; TyG, Triglyceride glucose; HOMA-IR, homeostasis model assessment of insulin resistance; HOMA-β, homeostasis model assessment of β-cell function; QUICKI, quantitative insulin sensitivity check index; HbA1c, hemoglobin A1C;

b Normally distributed values in the table are presented as the means ± SD, non-normally distributed values are presented as medians (25% and 75% interquartiles), and categorical variables are presented as frequencies(percentages). ANOVA for comparison of various samples with a normal distribution. Kruskal-Wallis test for abnormal distributions. *χ2* test for categorical variables.

†Bonferroni corrected *P* < 0.05, group1 and group2.

‡Bonferroni corrected *P* < 0.05, group2 and group3.

**Figure 1:**
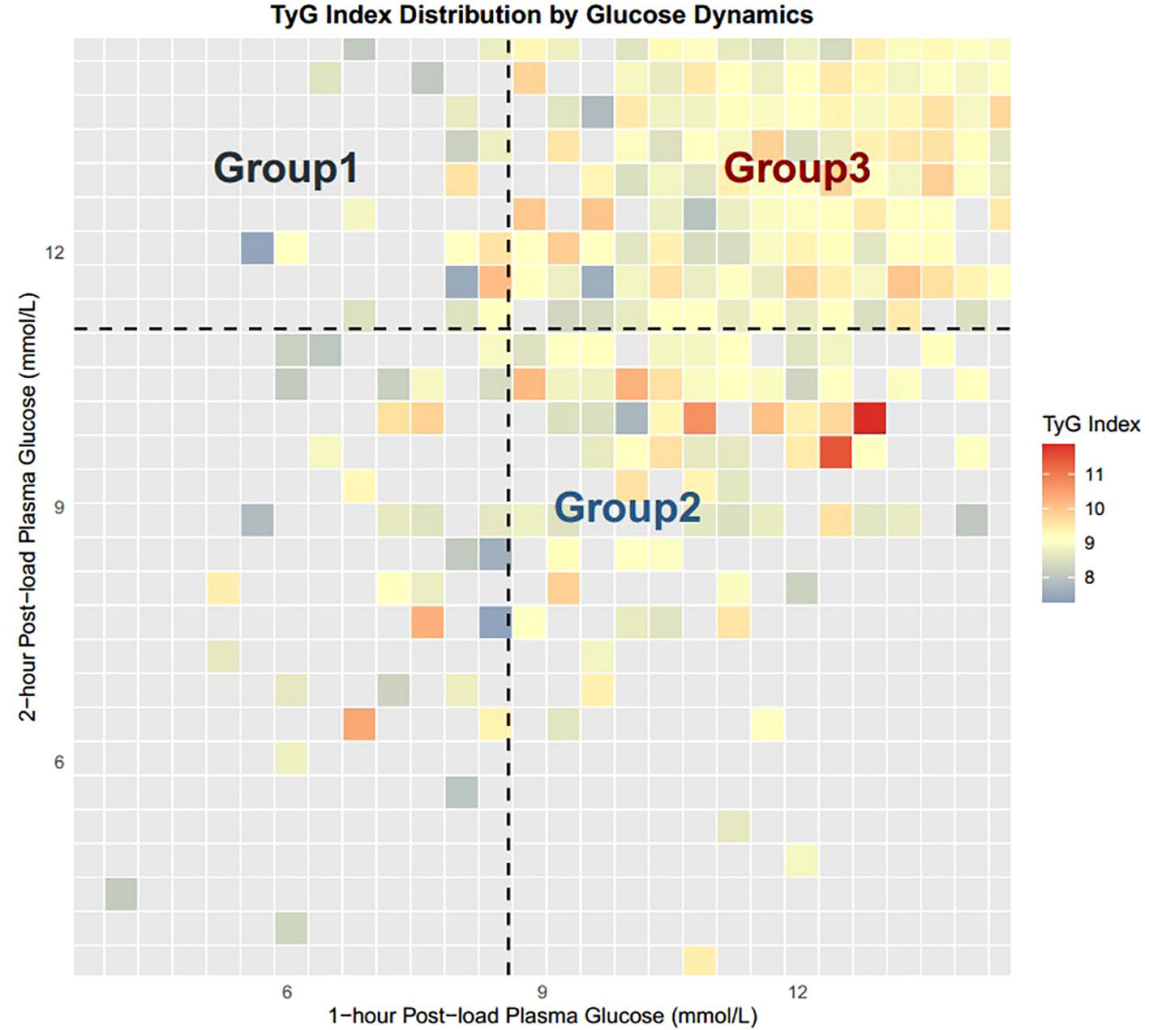
TyG index distribution by glucose dynamics.

3. As illustrated in **Figure 1**, a subset of individuals in Group 2 exhibited a significantly elevated TyG index compared to Group 3, indicating the presence of pronounced insulin resistance.

4. Based on TyG index thresholds of > 8.81 for males and > 8.73 for females to define higher insulin resistance, we stratified 1835 participants into the lower insulin resistance group (comprising 566 individuals) and the higher insulin resistance group (comprising 1269 individuals). A comparative analysis of general clinical characteristics and laboratory parameters between the two groups is presented in **Table 3**. Our findings revealed that, compared to the lower insulin resistance group, the higher insulin resistance group exhibited younger age, shorter disease duration, and a lower proportion of male participants. Additionally, the higher insulin resistance group demonstrated significantly elevated levels of SBP, DBP, BMI, ALT, AST, LDL, fasting C-peptide, 1-hour postprandial C-peptide, 2-hour postprandial C-peptide, 1h-PG,2h-PG and HbA1c, along with lower Scr levels. These findings indicate a stronger association of higher insulin resistance with adverse metabolic and laboratory parameters, highlighting the relevance of the TyG index as a marker for stratifying insulin resistance in this population.

**Table 3:** Comparisons of clinical variables between the lower insulin resistance and the higher insulin resistance group.

Variables	Lower insulin resistance	Higher insulin resistance	*x2/t/z*	*P*
n	566	1269	–	–
Age (y)	56.44 ± 12.65	53.34 ± 12.62 †	4.852	<0.001
Diabetes duration (y)	8.00 (2.00, 15.00)	6.00 (1.13, 12.00) †	-3.229	<0.001
Male (n, %)	(382, 67.49)	(838, 66.04) †	492.60	<0.001
SBP (mmHg)	134.37 ± 17.25	135.45 ± 17.09 †	-3.553	<0.001
DBP (mmHg)	78.94 ± 9.58	81.51 ± 10.75 †	-4.902	<0.001
BMI (kg/m^2^)	23.96 ± 3.40	25.91 ± 3.57 †	-10.936	<0.001
ALT (U/L)	16.00 (12.00, 24.00)	20.00 (14.00, 30.00) †	-5.532	<0.001
AST (U/L)	16.00 (14.00, 21.00)	17.00 (14.00, 23.00) †	-2.272	0.023
LDL (mmol/L)	2.50(1.97, 3.07)	2.78(2.16, 3.41) †	-5.790	<0.001
Scr (µmol/L)	58.00(48.28, 68.00)	56.80(45.85, 69.00) †	-2.071	0.038
C peptide (ng/mL)	0.86(0.43, 1.49)	1.58(1.02, 2.35) †	-15.203	<0.001
1-h C peptide (ng/mL)	2.31(1.18, 3.78)	3.10(1.94, 4.68) †	-7.519	<0.001
2-h C peptide (ng/mL)	3.64(1.90, 5.93)	4.41(2.65, 6.40) †	-4.804	<0.001
1-h PG	13.30(11.28, 15.33)	14.90(12.60, 17.20) †	-9.698	<0.001
2-h PG	15.20(12.70, 17.40)	16.20(13.80, 18.80) †	-6.318	<0.001
HbA1c(%)	8.30(6.80, 10.00)	8.80(7.50, 10.40) †	-5.149	<0.001

a SBP, systolic blood pressure; DBP, diastolic blood pressure; BMI, body mass index; ALT, Alanine aminotransferase; AST, Aspartate aminotransferase; LDL, low‐density lipoprotein; SCr, serum creatinine; 1h-PG, 1-hour post-load plasma glucose; 2h-PG, 2-hour post-load plasma glucose; HbA1c, hemoglobin A1C;

b Normally distributed values in the table are presented as the means ± SD, non-normally distributed values are presented as medians (25% and 75% interquartiles), and categorical variables are presented as frequencies(percentages). ANOVA for comparison of various samples with a normal distribution. Kruskal-Wallis test for abnormal distributions. χ2 test for categorical variables.

†Bonferroni corrected *P* < 0.05, lower insulin resistance group and higher insulin resistance group.

5. The results of univariate binary logistic regression analysis for T2DM with higher insulin resistance are presented in **Table 4**. Using the severity of insulin resistance as the dependent variable, 1h-PG and 2h-PG were separately included as independent variables to compare their respective impacts on insulin resistance. The analysis revealed that 1h-PG exhibited a stronger association with the TyG index than 2h-PG (*β* = 0.157, *OR* = 1.170, 95%*CI*: 1.133-1.209, *P* < 0.001 *vs*. *β* = 0.087, *OR* = 1.091, 95%*CI*:1.062-1.121, *P* < 0.001).

**Table 4:** Univariate binary logistic regression analysis the severity of insulin resistance as the dependent variable.

Variables	*β*	*OR*	*95%CI*	*P*
constant	-1.423	0.241	/	<0.001
1-h PG	0.157	1.170	1.133-1.209	<0.001
	*β*	*OR*	*95%CI*	*P*
constant	-0.570	0.566	/	<0.001
2-h PG	0.087	1.091	1.062-1.121	<0.001

**Figure 2:**
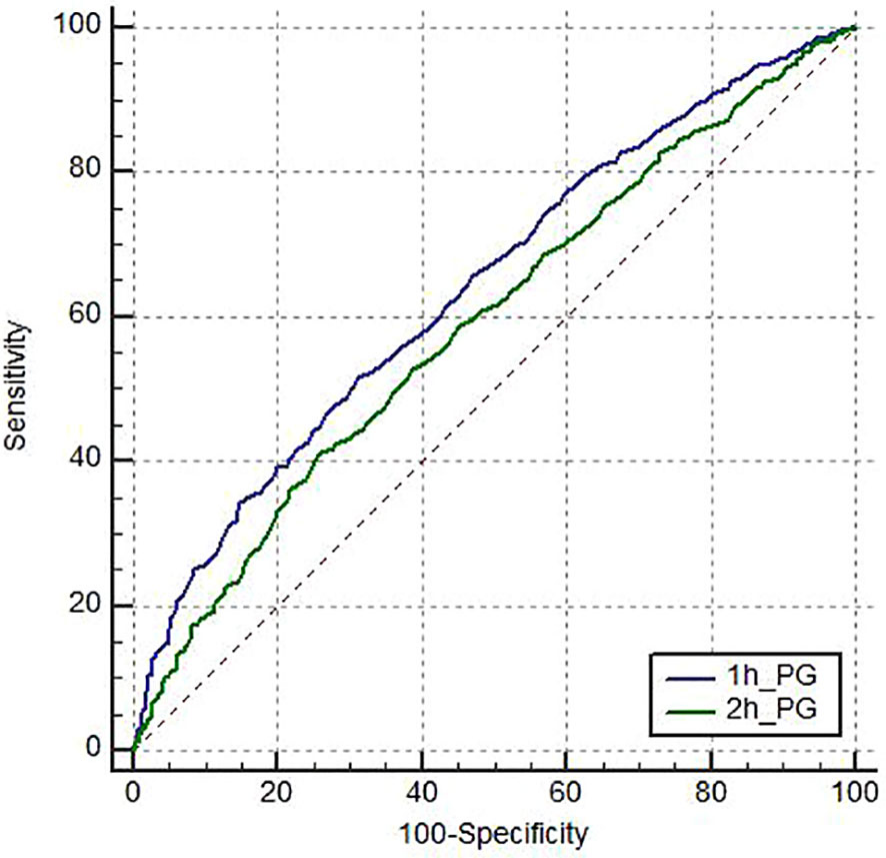
ROC curves of the 1h-PG, and 2h-PG for the prediction of insulin resistance.

6. ROC curve analysis was performed to evaluate predictive performance for insulin resistance. As illustrated in **Figure 2**, 1h-PG achieved a higher AUC than 2h-PG. Furthermore, as shown in **Table 5**, comparison of the AUCs using MedCalc software indicated a statistically significant difference between 1h-PG and 2h-PG (*Z* = 4.810, 95%*CI*: 0.0293-0.0697, *P* < 0.0001). These results indicate that 1h-PG provides superior predictive accuracy for insulin resistance compared to 2h-PG.

**Table 5:** Diagnostic performance of 1h-PG, and 2h-PG for insulin resistance in the total population.

Variables	AUC			Cut-off value	Sensitivity,%	Specificity,%
	*Est*.(95%*CI*)	*P*-value	*P* for comparison			
1h-PG	0.642.(0.619-0.664)	<0.001	–	14.7	51.5	68.9
2h-PG	0.592.(0.569-0.615)	<0.001	<0.001	17.2	41.2	74

## Discussion

Previous research has indicated a link between 1h-PG and insulin sensitivity in individuals with normal glucose tolerance (NGT). However, a critical gap exists because no studies have specifically investigated this association in patients already diagnosed with T2DM. Addressing this gap is important for improving early detection of insulin resistance and optimizing management strategies in this high-risk population. A major methodological challenge in T2DM is the potential confounding influence of chronic hyperglycemia, exogenous insulin therapy, and oral hypoglycemic agents, including secretagogues and insulin sensitizers, on conventional insulin resistance indices. These confounders can obscure true relationships when using HOMA-IR, HOMA-β, or QUICKI, particularly in patients with poor glycemic control. To overcome this limitation, our study employed the TyG index, a validated surrogate marker for insulin resistance that is less affected by pharmacological interventions and more feasible in clinical practice.

This study primarily aimed to determine whether 1h-PG exhibits a more robust association with the TyG index than 2h-PG. We observed that 1h-PG showed a stronger correlation with the TyG index (*r* = 0.273) compared to 2h-PG (*r* = 0.173, *P* < 0.001). Logistic regression analysis (*β* = 0.157, *OR* = 1.170, *95%CI*: 1.133–1.209, *P* < 0.001 *vs*. *β* = 0.087, *OR* = 1.091, *95%CI:* 1.062–1.121, *P* < 0.001) and ROC curve evaluation (*AUC* 0.642 *vs*. 0.592, *P* < 0.001) further confirmed this finding, indicating that 1h-PG may serve as a more robust and clinically practical indicator of insulin resistance in T2DM patients.

Metabolic syndrome, including obesity, diabetes, NAFLD, and cardiovascular diseases, is clinically characterized by insulin resistance, which plays a central role in its pathophysiology. Accurate assessment of insulin resistance is therefore essential for diagnosis, risk stratification, and therapeutic decision-making. The hyperinsulinemic-euglycemic clamp technique (HIET), developed by DeFronzo et al., which directly quantifies insulin sensitivity using precise infusion pumps and computer technology, remains the gold-standard methodology for precise insulin resistance quantification (13). This method yields stable and reproducible results, however, widespread clinical adoption is hindered by three key factors: (1) technically demanding protocols, (2) time-intensive procedures (~4–6 hours), and (3) significant cost burdens. Fasting state indices, including the HOMA for estimating insulin resistance (HOMA-IR) and β-cell function (HOMA-β), as well as the QUICKI, are commonly used to evaluate IR (14, 15). While HOMA-IR, HOMA-β, and QUICKI can accurately describe IR in individuals with NGT, their application in individuals with impaired glucose regulation, including diabetic patients, particularly those with poor glycemic control, requires caution due to the pronounced “glucotoxicity” that suppresses β-cell function. Additionally, these indices are challenging to implement in resource-limited clinical settings and are influenced by exogenous insulin administration, especially in small sample sizes. Furthermore, the use of insulin sensitizers and insulin secretagogues may interfere with HOMA-IR, HOMA-β, and QUICKI measurements, potentially affecting the accurate assessment of true insulin resistance in patients (16). Thus, there is a clear need for a practical, reliable, and less confounded marker of insulin resistance that can be applied broadly in clinical and research settings, motivating the investigation of alternative indices such as the TyG index.

The TyG index has emerged as a valuable biomarker in the assessment of insulin resistance, which is a key underlying mechanism in T2DM and its complications. Accumulating evidence demonstrates a robust association between the TyG index and incident T2DM, supporting its role as an independent predictive marker for the disease (17). Nevertheless, challenges remain in identifying individuals at risk for diabetes and its complications at an early stage.

Moreover, higher TyG index levels are associated with the incidence and progression of diabetic complications, including retinopathy and nephropathy. Despite its validated predictive value, widespread adoption of the TyG index in clinical practice is limited by the need for standardization and clear guidance on cutoff values for different populations.

Specifically, the TyG index is significantly correlated with diabetes-related mortality and can predict cardiovascular death in individuals with metabolic syndrome (18). It also reflects long-term glycemic control more accurately than conventional measures, capturing glycemic variability in T2DM populations (mean correlation *r* = 0.62 with 12-month HbA1c) (19). Moreover, higher TyG levels are associated with the occurrence of diabetic complications. Yao et al. established that higher TyG index levels significantly predict both diabetic retinopathy incidence (*HR* 1.58, 95% *CI* 1.32-1.89) and progression to vision-threatening stages (OR 2.14) (20). Similarly, LV et al. established a significant association between higher TyG index levels and both incident proteinuria (*OR* 1.42, 95% *CI* 1.18-1.71) and accelerated glomerular filtration rate decline (-3.2 mL/min/year per SD increase) in diabetic cohorts (21). In 2008, the TyG index was first reported as a potential surrogate marker for HOMA-IR in diagnosing IR in apparently healthy subjects (3). Subsequent studies comparing the TyG index with the current gold standard, the hyperinsulinemic-euglycemic clamp (HIEC), have demonstrated that the TyG index is an optimal tool for assessing IR, exhibiting high sensitivity (96.5%) and specificity (85%). Additionally, this index is characterized by its ease of acquisition, low cost, and high feasibility, making it a practical and accessible alternative for clinical and research applications (2). Despite its validated predictive value, widespread adoption of the TyG index in clinical practice is limited by the need for standardization and clear guidance on cutoff values for different populations.

In parallel, conventional postprandial glucose assessment relies heavily on 2h-PG levels, which are important but may fail to detect early abnormalities in glucose metabolism. Emerging evidence increasingly highlights the clinical significance of 1h-PG, suggesting that elevated 1h-PG represents an earlier stage of dysglycemia, preceding abnormalities detectable by 2h-PG. Individuals with elevated 1h-PG within the NGT range show increased glycemic variability, impaired β-cell function, insulin resistance, elevated blood pressure, and dyslipidemia, indicating substantial pathophysiological changes even before overt hyperglycemia (22, 23). Several mechanistic and clinical studies have confirmed that 1h-PG correlates more strongly with β-cell dysfunction than 2h-PG. For instance, pathophysiological analyses using HIEC and the intravenous glucose tolerance test (IVGTT) techniques demonstrated that individuals with elevated 1h-PG exhibit lower insulin sensitivity and impaired β-cell function compared to those with normal 1h-PG (24). Observational studies in Native Americans and other populations further revealed that 1h-PG outperforms 2h-PG in predicting acute insulin secretion and β-cell glucose sensitivity (25). These findings collectively suggest that relying solely on 2h-PG may delay early detection of insulin resistance and β-cell dysfunction, potentially limiting opportunities for timely intervention (8, 26). Therefore, there is a clear unmet need to incorporate 1h-PG assessment alongside robust indices such as the TyG index to identify high-risk individuals earlier and more accurately. Utilizing 1h-PG for screening, particularly in conjunction with the TyG index, provides a time- and cost-efficient strategy for early detection of individuals at risk for T2DM and related complications, thereby facilitating timely intervention and potentially preventing progression to overt disease and its associated complications (27).

However, to date, no studies have explored the relationship between 1h-PG and β-cell insulin sensitivity in individuals already diagnosed with T2DM, which constitutes the primary focus of our investigation. Considering the chronic hyperglycemia-induced glucotoxicity in these patients, as well as the potential confounding effects of insulin, insulin sensitizers, and insulin secretagogues on traditional insulin resistance indices such as HOMA-IR, HOMA-β, and QUICKI, we chose to concentrate on the recently proposed novel index, the TyG index. Our study enrolled all of 1835 individuals already diagnosed with T2DM, who were stratified into three groups based on the following criteria: 1h-PG ≥ 8.6 mmol/L and 2h-PG ≥ 11.1 mmol/L following a steamed buns meal test. A statistically significant increase in the TyG index was observed in Group 2 compared with Group 1. Although Group 3 showed a trend toward a higher TyG index compared with Group 2, the difference did not reach statistical significance. Furthermore, using TyG index cutoffs of > 8.81 for males and > 8.73 for females to define higher insulin resistance, we divided the 1835 participants into the lower insulin resistance and the higher insulin resistance groups. We observed that the higher insulin resistance group exhibited significantly higher 1h-PG levels compared with the lower insulin resistance group. Spearman bivariate correlation analysis demonstrated that the correlation between 1h-PG and the TyG index (*r* = 0.273) was stronger than that between 2h-PG and the TyG index (*r* = 0.173). Univariate binary logistic regression analysis further confirmed that the association between 1h-PG and the TyG index (*β* = 0.157, *OR* = 1.170, 95% *CI*: 1.133–1.209, *P* < 0.001) was stronger than that between 2h-PG and the TyG index (*β* = 0.087, *OR* = 1.091, 95% *CI*: 1.062–1.121, *P* < 0.001). Additionally, ROC curve analysis yielded consistent conclusions, supporting the superior predictive value of 1h-PG for insulin resistance compared with 2h-PG. In this study, both 1h-PG and 2h-PG showed positive correlations with the TyG index, although the magnitudes of these correlations were modest. However, even modest correlation coefficients may still carry meaningful clinical implications, particularly in the context of early metabolic deterioration. The slightly stronger correlation observed for 1h-PG compared with 2h-PG suggests that early postprandial glucose excursion may better capture subtle impairments in insulin sensitivity and β-cell dysfunction. This is consistent with previous studies indicating that 1h-PG reflects early dysglycemia more sensitively than traditional 2-hour measurements. Moreover, despite the modest numerical difference in correlation strength, the superiority of 1h-PG was further supported by logistic regression and ROC analyses, where 1h-PG demonstrated significantly better predictive performance for insulin resistance. Taken together, these findings indicate that even small improvements in predictive markers can translate into enhanced clinical utility, reinforcing the potential value of 1h-PG as an accessible and cost-effective indicator of insulin resistance.

It is noteworthy that, as shown in **Table 2**, compared to Group 1, Group 2 exhibited a significant increase in1-hour postprandial C-peptide levels following the steamed bread meal test, indicating early β-cell dysfunction and a compensatory elevation in insulin secretion. In contrast to Group 2, Group 3 demonstrated lower fasting C-peptide levels, as well as reduced 1-hour and 2-hour postprandial C-peptide values, highlighting that β-cell function further deteriorates and endogenous insulin secretion declines with disease progression. Additionally, **Table 2** reveals that there were no statistically significant differences in HOMA-IR and QUICKI values among the three groups. This discrepancy raises a methodological concern: traditional indices like HOMA-IR and QUICKI may not accurately reflect insulin resistance in certain populations. Specifically, the HOMA model has inherent limitations, particularly in lean individuals with metabolic risk factors, as emphasized by Kang et al, (28) which are commonly observed in Asian populations. Furthermore, some studies suggest that HOMA-IR may underestimate true insulin resistance in these populations (29), indicating a need for alternative markers such as the TyG index.

This investigation has important limitations warranting discussion. First, the single-center design and exclusive inclusion of hospitalized T2DM patients may restrict the external validity of these findings, particularly regarding outpatient populations and broader geographic representation. As a result, the conclusions may not fully capture the characteristics of real-world patients across diverse settings. Second, although the TyG index was selected to mitigate the potential confounding effects of glucotoxicity, exogenous insulin, and oral hypoglycemic agents, we acknowledge that medication use—particularly metformin, insulin, and other agents that may influence lipid and glucose metabolism—was not adjusted for in our statistical analyses. Therefore, residual confounding related to antidiabetic therapies cannot be excluded. Third, as a cross-sectional study, this analysis is unable to establish a causal relationship between the TyG index and 1h-PG. Future investigations should adopt (i) multicenter collaborative designs, (ii) adequately powered sample sizes (n > X,000), and (iii) prospective longitudinal follow-up to determine the predictive validity of 1h-PG for insulin resistance.

## Conclusion

This study primarily elucidates the relationship between the TyG index and 1h-PG in individuals already diagnosed with T2DM. Compared to 2h-PG, 1h-PG exhibits a stronger association with insulin resistance, as assessed by the TyG index in these patients. Its high efficiency (requiring only a 1-hour test) and superior sensitivity (earlier detection than conventional markers) support its preferential application in clinical screening.

## Data Availability

The raw data supporting the conclusions of this article will be made available by the authors, without undue reservation.
